# Effects of eszopiclone on safety, subjective measures of efficacy, and quality of life in elderly and nonelderly Japanese patients with chronic insomnia, both with and without comorbid psychiatric disorders: a 24-week, randomized, double-blind study

**DOI:** 10.1186/1744-859X-11-15

**Published:** 2012-06-25

**Authors:** Naohisa Uchimura, Atsushi Kamijo, Takao Takase

**Affiliations:** 1 Department of Neuropsychiatry, Kurume University School of Medicine, 67 Asahi-machi, Kurume, 830-0011, Japan; 2 Eisai Co., Ltd., Tokyo, Japan

**Keywords:** Elderly, Long-term, Eszopiclone, Insomnia, Psychiatric disorder, Quality of life, Japanese

## Abstract

**Background:**

The primary objective of this study was to evaluate long-term (24-week) safety of eszopiclone in elderly and nonelderly Japanese patients with chronic insomnia. The secondary objectives were to evaluate short-term (4-week) efficacy and to assess for rebound insomnia or dependence after long-term treatment.

**Methods:**

Patients (n = 164 elderly; n = 161 nonelderly), with or without psychiatric comorbidities, were randomized to receive low-dose (1 mg, elderly; 2 mg, nonelderly) or high-dose (2 mg, elderly; 3 mg, nonelderly) eszopiclone. The safety evaluation included adverse events, vital signs, clinical laboratory parameters, and electrocardiogram. Efficacy was assessed using patient reports of sleep latency (SL), total sleep time (TST), wake time after sleep onset (WASO), number of awakenings (NA), quality of sleep, depth of sleep, daytime sleepiness, daytime ability to function, and the 36-item Short Form (SF-36) Health Survey.

**Results:**

The rate of adverse events was 81.5% in the 1-mg elderly group, 79.5% in the 2-mg elderly group, 82.1% in the 2-mg nonelderly group, and 87.0% in the 3-mg nonelderly group. Dysgeusia was the most common adverse event and was dose-related. Of 12 serious adverse events, none were considered by the investigator to be related to study medication. No rebound insomnia was observed. Eszopiclone significantly improved SL, TST, WASO, NA, and daytime sleepiness and function from baseline to Week 4, irrespective of age and psychiatric comorbidity. Improvements were also observed in SF-36 Mental Health Component scores in elderly and nonelderly patients with psychiatric comorbidities.

**Conclusions:**

Irrespective of age, eszopiclone appeared safe as administered in this study for 24 weeks. Eszopiclone improved sleep variables in insomnia patients with and without psychiatric disorders and health-related quality of life in those with psychiatric disorders.

**Trial registration:**

ClinicalTrials.gov #NCT00770692; http://clinicaltrials.gov/ct2/show/NCT00770692.

## Background

Epidemiologic surveys from different countries suggest that 30% of adults have 1 or more symptoms of insomnia [1], and an estimated 10% of individuals exhibit symptoms of functional impairment during the day that is consistent with insomnia [2]. In the Japanese population, insomnia affects 17.3% to 22.3% of men and 20.5% to 21.5% of women [3]. Psychotropic medications are often used for management of insomnia [4]. These medications, however, may be associated with an increased risk of falls among the elderly [5]. Eszopiclone is an (S)-isomer of zopiclone that is structurally classified as a nonbenzodiazepine hypnotic [6,7]. In 2004, eszopiclone was approved by the US Food and Drug Administration for the treatment of insomnia in elderly and nonelderly adults [6,8]. The clinical studies used for registration in the United States included both short- and long-term studies but did not include long-term studies in elderly patients [9-13]. Overall outcomes in these studies showed that eszopiclone significantly reduced sleep latency (SL), increased total sleep time (TST), reduced wake time after sleep onset (WASO), and was generally well tolerated compared with placebo [8-13].

Increasing age has been identified as a risk factor for insomnia. As the population ages, the number of elderly patients with insomnia is expected to increase, along with the need for safe and effective treatment options. Insomnia in elderly patients often presents comorbidly with medical or psychiatric conditions that usually require chronic therapy [14]. In a 12-week study of elderly patients (N = 388) with either primary or comorbid chronic insomnia, patient-reported sleep and daytime function significantly improved following treatment with eszopiclone compared with placebo, with no evidence of rebound insomnia or withdrawal symptoms after treatment discontinuation [15]. The rates of dizziness (4.1% and 1.5%) and falls (1.0% and 0.5%) were relatively low in patients receiving eszopiclone and placebo, respectively, and eszopiclone was generally well tolerated and safe in this patient population [15]. Assessment of the tolerability, safety, and efficacy of eszopiclone beyond 12 weeks has not been reported in elderly patients.

Eszopiclone has been shown to be efficacious for primary insomnia in both elderly [12,13,15] and nonelderly patients [9,11,16,17]. Furthermore, the effects of eszopiclone on sleep variables and comorbid conditions were reported in nonelderly patients with insomnia comorbid with depression, major depressive disorder, and generalized anxiety disorder [18-20]. No study has compared treatment of eszopiclone for insomnia in patients with and without psychiatric comorbidities. Although US studies have established the safety and efficacy of eszopiclone in elderly and nonelderly adults with chronic insomnia [9,11-13,15-17], there have been no reports on the safety and efficacy of eszopiclone in Japanese populations.

The primary objective of the current study was to evaluate the safety of eszopiclone, administered over 24 weeks, in Japanese elderly and nonelderly patients with chronic insomnia. Assessment of safety included adverse events, vital signs, clinical laboratory parameters, electrocardiogram (ECG), and Questionnaire of Drug Dependence. Secondary objectives were to evaluate the efficacy of eszopiclone over 4 weeks in patients with insomnia with or without psychiatric disorders and to evaluate the presence or absence of sleep rebound and dependence after long-term treatment with eszopiclone. Measures of efficacy included SL, TST, WASO, number of awakenings (NA), quality of sleep, depth of sleep, daytime sleepiness, daytime ability to function, and the 36-item Short Form (SF-36) Health Survey. When this study was planned, a placebo arm for chronic insomnia did not seem to be acceptable from an ethical standpoint and in light of the medical environment in Japan. The safety of eszopiclone instead was evaluated by comparison between pre- and post-treatment data.

## Methods

This multicenter, randomized, double-blind, parallel-group study was conducted at 46 sites in Japan from October 2008 to May 2010. The protocol was approved by local institutional review boards, and the study was conducted in accordance with the principles of the Declaration of Helsinki and Japan Good Clinical Practice. All patients signed written informed consent prior to study entry.

### Patient selection

Outpatients 20 to 84 years of age seeking evaluation and treatment for their sleep difficulties were considered eligible for study entry when all of the following inclusion criteria were met: (1) presence of primary insomnia, as diagnosed by the *Diagnostic and Statistical Manual of Mental Disorders,* Fourth Edition, Text Revision (DSM-IV-TR) [21], or insomnia associated with a physical or psychiatric disorder; (2) presence of reported symptoms with SL ≥30 minutes on 3 or more nights per week and TST ≤390 minutes on 3 or more nights per week for 4 weeks or more prior to screening; and (3) presence of symptoms with SL ≥30 minutes on 3 or more nights (including at least 2 consecutive nights) and TST ≤390 minutes on 3 or more nights (including at least 2 consecutive nights) as recorded in the patient self-report sleep diary during the screening period. The threshold of TST ≤390 minutes and the criterion of 2 consecutive nights were used to ensure that the patient population had a level of insomnia severity likely to show a treatment effect.

Patients were excluded if they met any of the following criteria: (1) risk of suicide, manic episode, post-traumatic stress disorder (PTSD), history of or current alcohol dependence or abuse, history of or current drug dependence or abuse, anorexia nervosa, bulimia nervosa, or antisocial personality disorder, as diagnosed by the Mini-International Neuropsychiatric Interview (M.I.N.I.) Japanese version 5.0.0; (2) drug-induced insomnia; (3) primary sleep disorders other than primary insomnia (eg, circadian rhythm disorder, restless leg syndrome, periodic limb movement, sleep apnea); (4) severely disturbed sleep by chronic pain, fever, diarrhea, frequent urination, or coughing; (5) organic psychiatric disorder; (6) suicidal ideation or attempt in the past 5 years; (7) clinically severe dysfunction of the liver, kidney, cardiovascular system, or hematologic system or presence of a malignant tumor; and (8) pregnancy or breast-feeding.

The restricted concomitant medications from 2 weeks before to 4 weeks after starting eszopiclone administration included sedative hypnotics, anxiolytics not indicated for insomnia, neurologic disease medications, antiepileptics, Parkinson’s disease medications, antihistamines, analgesics, adrenal corticosteroids, bronchovasodilators, melatonin, oriental medicines indicated for insomnia, and herbal preparations or supplements used for insomnia. The prohibited concomitant medications from Day −10 to the end of study included sedative hypnotics other than eszopiclone, anxiolytics indicated for insomnia, and potent inhibitors and inducers of cytochrome P450 isoenzyme 3A4.

### Study procedures

**Figure 1:**
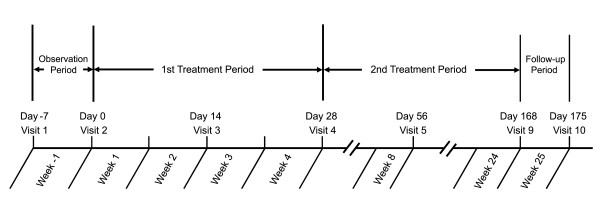
**Summary of study schematic from screening through study completion.** Visits 6, 7, and 8 were conducted at 4-week intervals. Adverse events were collected from Weeks 1 to 25. Sleep variables were obtained at Week −1 (baseline) and Weeks 1 to 4. SF-36 was assessed at Weeks −1, 4, 12 and 24.

The study consisted of 10 visits and 4 periods: the screening period (Week −1; baseline), the first treatment period (Weeks 1 to 4), the second treatment period (Weeks 5 to 24), and the follow-up period (Week 25) (Figure 1). At Day −7, patients signed written informed consent, had their baseline characteristics (demographics, medications, and sleep, medical, and psychiatric histories) recorded, and completed all other screening procedures, including vital signs, ECG, and clinical laboratory tests.

Patients who met inclusion criteria to enter into the screening period were instructed to complete a self-report sleep diary for 1 week. Patients used the diary to record various sleep parameters, such as SL, TST, and WASO. At Day 0, SL and TST for the previous 7 days were assessed to judge the eligibility for study treatment. The principal investigators and sub-investigators were able to make a clinical diagnosis of primary insomnia, insomnia associated with a physical disorder, or insomnia associated with a psychiatric disorder by taking underlying physical conditions and psychiatric conditions into consideration.

Drug allocation was performed by stratified block randomization with 4 strata (elderly patients with psychiatric disorders, elderly patients without psychiatric disorders, nonelderly patients with psychiatric disorders, nonelderly patients without psychiatric disorders). A third-party drug randomization manager randomly assigned the study medications to the eszopiclone 1-mg group and the 2-mg group for each of the 2 elderly patient strata and to the eszopiclone 2-mg group or the 3-mg group for each of the 2 nonelderly patient strata, according to the treatment allocation table (key code list) prepared by him/herself. The drug randomization manager sealed drug codes immediately after randomizing patients and kept the drug codes until code-break.

Eligible patients with or without a comorbid psychiatric disorder were randomized by a third-party enrollment center in a double-blind manner to 1 mg or 2 mg eszopiclone (elderly patients, 65 to 84 years of age) or to 2 mg or 3 mg eszopiclone (nonelderly patients, 20 to 64 years of age). The use of different doses for nonelderly (2 mg or 3 mg) and elderly patients (1 mg or 2 mg) was based on the pharmacokinetic profile of eszopiclone in studies of healthy subjects and on the findings of previous clinical studies. Study medication was dispensed at the study site with instructions on proper administration and was self-administered by patients at home. Patients took the assigned dose of eszopiclone at bedtime starting from Day 0 and were instructed to fill out a self-report diary to assess sleep variables every day during the first treatment period.

An uptitration was permitted for patients whose insomnia did not improve after 4 weeks of treatment with low-dose eszopiclone. At the end of the first treatment period (Week 4), the option of a 1-mg uptitration for the second treatment period was evaluated for patients meeting all of the following criteria: (1) no improvement in SL or TST when comparing values at Week 4 and baseline (ie, baseline SL ≤ Week 4 SL, baseline TST ≥ Week 4 TST), (2) patient rating of “not changed” or “worsened” on the global impression of overall improvement of sleep, and (3) investigators’ judgment regarding the safety of the dose increase. Patients who were already taking the maximum dose (ie, 2 mg in the elderly group and 3 mg in the nonelderly group) received an additional 1-mg placebo tablet, and eligible patients who had been treated initially with the lower dose of eszopiclone (ie, 1 mg in the elderly group and 2 mg in the nonelderly group) received an additional 1-mg eszopiclone tablet during the second treatment period. Patients not eligible for uptitration continued to receive their previous dose of study drug. Although patients and investigators remained blinded to assigned dose, the study investigator may have apprehended the dose given after dose escalation.

Patients underwent scheduled examinations at all visits and clinical laboratory tests at Weeks −1 (screening), 4, 8, 12, 16, and 20 and at Week 24 or final visit due to early discontinuation. The investigators collected information on adverse events from the time of the first dose of eszopiclone and determined the relationship of all reported adverse events as “related” or “not related” to administration of eszopiclone. The follow-up period lasted for 1 week from the end of the treatment period or the day on which treatment was discontinued. During the follow-up period, post-treatment safety, including sleep rebound and dependency, was evaluated.

### Study assessments

Adverse events were recorded at all study visits except screening and were rated by study investigators for intensity (mild, moderate, severe), seriousness, and relationship to study medication (unrelated, possibly related, probably related). Vital signs, including blood pressure and heart rate, were collected at Week −1 through the final visit, and clinical laboratory assessments (including hematology, blood biochemistry, and urinalysis) were performed at screening and Week 4 through the final visit. An ECG was obtained at screening and repeated at Week 4 and at the final visit. The Questionnaire of Drug Dependence was administered at the end of the follow-up period (Week 25 or the week following the final visit) [22]. Rebound insomnia was defined as worsening in SL, TST, or WASO after eszopiclone discontinuation compared with baseline and was assessed at the follow-up visit using a patient-reported sleep diary. Worsening was defined as an increase in median SL or WASO or a decrease in median TST at follow-up compared with baseline.

Efficacy assessments were the change in patient-reported data from baseline to Week 4 for SL, TST, WASO, NA, quality of sleep, depth of sleep, daytime sleepiness, and daytime ability to function. Quality of sleep, depth of sleep, and daytime ability to function were assessed based on the patient-reported sleep diary using a numeric rating scale, with scores ranging from 0 (poor) to 10 (excellent); daytime sleepiness was similarly rated and was scored from 0 (very sleepy) to 10 (wide awake). Change from baseline to Week 24 on the SF-36 Health Survey Version 2-Japanese Version was assessed to evaluate health-related quality of life.

### Statistical methods

The study was powered at N = 320 to achieve a >80% probability of detecting ≥1 adverse event at a 0.5% rate and to meet the criteria for safety evaluation in accordance with the International Conference on Harmonization of Technical Requirements for Registration of Pharmaceuticals for Human Use (ICH E1) guideline, *The Extent of Population Exposure to Assess Clinical Safety for Drugs Intended for Long-term Treatment of Non-Life-Threatening Conditions*.

The safety analysis population included patients who received ≥1 dose of study medication and ≥1 safety evaluation. The efficacy analysis population comprised all patients who had ≥1 dose of study medication and ≥1 efficacy evaluation. Most statistical analyses were performed using SAS software (Version 9.1.3). All statistical tests were conducted at the 15% (2-tailed) significance level for homogeneity of background among each allocation and at the 5% (2-tailed) significance level for comparison of other evaluation items.

For continuous demographic and baseline variables, summary statistics were calculated for elderly and nonelderly patients in each dose group. The number and proportion of patients per subgroup were calculated according to elderly and nonelderly groups for ordered categorical variables and categorical variables. A *t*-test was performed for continuous variables and Fisher exact test for categorical variables to confirm the homogeneity of stratification factors (ie, 1 mg/2 mg in elderly patients and 2 mg/3 mg in nonelderly patients; 1 mg/2 mg with psychiatric disorders in elderly patients, 1 mg/2 mg without psychiatric disorders in elderly patients, 2 mg/3 mg with psychiatric disorders in nonelderly patients, and 2 mg/3 mg without psychiatric disorders in nonelderly patients).

The number of adverse events, the number of patients with adverse events, and adverse event rates were obtained by group for elderly and nonelderly patients with and without psychiatric disorders. For summaries of adverse event rates, the number of patients who reported ≥1 adverse event and the percentage of patients in the safety analysis set were classified according to the Medical Dictionary for Regulatory Activities (Japanese version 13.0), system organ class, and preferred term as well as by the investigator’s assessment of relationship to drug and severity. For blood pressure, heart rate, and clinical laboratory assessments, summary statistics of data and changes at each evaluation time were calculated by group for continuous variables, whereas frequency and percentage were calculated for categorical variables. The frequency distribution of presence/absence of abnormal changes and the percent change were calculated in each group (elderly and nonelderly, with or without psychiatric disorders) for abnormal changes in blood pressure, heart rate, clinical laboratory tests, and ECG parameters. For each item of the Questionnaire of Drug Dependence, the frequency distribution and percentage were calculated for elderly and nonelderly patients with and without psychiatric disorders.

Summary statistics for sleep variables (SL, TST, WASO, and NA) were calculated by dose group for elderly and nonelderly patients with and without psychiatric disorders. For sleep variables (SL, TST, WASO, and NA), the median of 7 days in the week of Weeks −1 (baseline), 1, 2, 3, and 4 was used as the representative value for subgroups (ie, elderly and nonelderly with and without psychiatric disorders). For statistical testing in each subgroup, paired *t* tests (using log-transformed data) were performed to compare Weeks 1 to 4 with baseline. An analysis of variance model with stratification for presence/absence of psychiatric disorders was used for inter-group comparisons (1 mg vs 2 mg in elderly patients; 2 mg vs 3 mg in nonelderly patients). For quality of sleep, depth of sleep, daytime sleepiness, and daytime ability to function, summary statistics were calculated and paired *t* tests were performed to compare Weeks 1 to 4 with baseline. Summary statistics were calculated for subscale and summary score of the SF-36 for elderly and nonelderly patients with and without psychiatric disorders. The SF-36 scores were transformed to norm-based scorings representing the national standard value of Japanese people (mean, 50; standard deviation [SD], 10). The norm-based scorings at Week 24 or final visit were compared with baseline using paired *t* tests, with the last observation carried forward (LOCF). Because the safety evaluation was the primary objective of the study, adjustment for multiple comparisons was not performed.

To assess potential rebound insomnia, SL, TST, and WASO in the follow-up period (Week 25) were compared with baseline using paired *t* tests of log-transformed data. Rebound insomnia was considered to have occurred if the sleep parameters clearly deteriorated after the completion or discontinuation of study treatment.

## Results

### Patients

**Figure 2:**
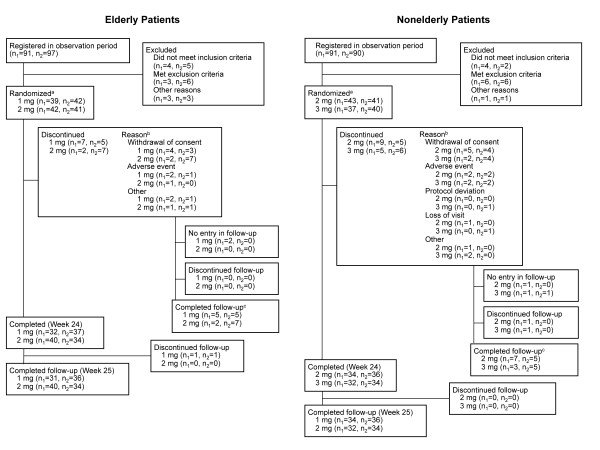
**Patient disposition.**^a^Randomization and the first administration of eszopiclone were performed on Day 0; ^b^Patients could have more than 1 reason for study discontinuation; ^c^During the week after discontinued intervention. n_1_ = number of patients with comorbid psychiatric disorders; n_2_ = number of patients without psychiatric disorders.

Of 369 patients screened, 164 elderly patients (81 with psychiatric disorders, 83 without psychiatric disorders), and 161 nonelderly patients (80 with psychiatric disorders, 81 without psychiatric disorders) were randomized to receive eszopiclone (Figure 2). All of the 164 elderly patients and the 161 nonelderly patients who were enrolled in the treatment period were included in the safety analysis set. The efficacy evaluation set included all of the elderly and nonelderly patients who were enrolled in the treatment period, excluding 1 elderly patient in the 1-mg group (with psychiatric disorders) who had no evaluable efficacy data. A total of 143 (87.2%) patients in the elderly group and 136 (84.5%) patients in the nonelderly group completed treatment with eszopiclone. Reasons for discontinuation are described in Figure 2.

Demographics and baseline values for sleep variables were similar in each subgroup (Table 1). There were no statistically significant (*P* < 0.15) differences in baseline sleep variables between patients assigned each of the 2 doses of eszopiclone, with the following exceptions: TST for 2 mg versus 3 mg in nonelderly patients with psychiatric disorders (mean, 296.5 vs 315.3 min; *P* < 0.15), and TST for 2 mg versus 3 mg in nonelderly patients without psychiatric disorders (284.7 vs 301.6 min; *P <* 0.15).

**Table 1:** Demographic characteristics and baseline values for sleep variables

**Characteristic**	**Elderly**		**Elderly**		**Nonelderly**		**Nonelderly**	
	**1 mg**		**2 mg**		**2 mg**		**3 mg**	
	**With**^**a**^	**Without**^**b**^	**With**^**a**^	**Without**^**b**^	**With**^**a**^	**Without**^**b**^	**With**^**a**^	**Without**^**b**^
	**(n = 38)**	**(n = 42)**	**(n = 42)**	**(n = 41)**	**(n = 43)**	**(n = 41)**	**(n = 37)**	**(n = 40)**
Age, y, mean (SD)	71.5 (5.0)	69.5 (3.9)	70.3 (5.1)	71.0 (4.4)	37.3 (10.7)	43.0 (10.1)	37.6 (9.1)	45.9 (12.2)
Sex, n (%)								
Male	20 (52.6)	15 (35.7)	20 (47.6)	14 (34.1)	11 (25.6)*	18 (43.9)	22 (59.5)	19 (47.5)
Female	18 (47.4)	27 (64.3)	22 (52.4)	27 (65.9)	32 (74.4)*	23 (56.1)	15 (40.5)	21 (52.5)
Body mass index, kg/m^2^, mean (SD)	22.8 (3.4)	23.1 (3.1)	23.1 (3.3)	23.5 (3.3)	22.6 (4.2)	24.0 (4.2)	23.3 (4.0)^†^	22.5 (3.2)
Sleep latency, min								
Mean (SD)	63.3 (44.0)	67.4 (37.4)	71.8 (49.6)	69.7 (45.1)	78.5 (62.8)	64.8 (41.3)	65.7 (46.0)	62.4 (38.9)
Median	60.0	60.0	60.0	60.0	60.0	60.0	60.0	55.0
Total sleep time, min								
Mean (SD)	317.6 (77.1)	311.1 (70.4)	310.2 (67.2)	305.6 (52.4)	296.5 (46.8)^‡^	284.7 (40.6)^†^	315.3 (60.5)	301.6 (54.9)
Median	330.0	310.0	330.0	300.0	300.0	290.0	330.0	300.0
Wake time after sleep onset, min								
Mean (SD)	66.7 (56.9)	57.1 (48.1)	65.6 (63.0)	72.1 (63.6)	51.7 (41.5)	54.9 (42.6)	40.8 (40.5)	43.8 (41.6)
Median	60.0	40.0	35.0	50.0	40.0	60.0	30.0	33.8

^a^ Insomnia with comorbid psychiatric disorders.

^b^ Insomnia without comorbid psychiatric disorders.

*P < 0.01 vs nonelderly patients with psychiatric disorders receiving 3 mg eszopiclone.

^†^*P* < 0.05 vs nonelderly patients without psychiatric disorders receiving 3 mg eszopiclone.

^‡^*P* < 0.05 vs nonelderly patients with psychiatric disorders receiving 3 mg eszopiclone.

SD = standard deviation.

Approximately half of enrolled patients had psychiatric disorders (49.4% of elderly patients and 49.7% of nonelderly patients). The most common psychiatric disorders among patients with insomnia associated with a psychiatric disorder were major depressive disorder, generalized anxiety disorder, and dysthymic disorder (Table 2). In insomnia patients without psychiatric disorders, 76 of 83 elderly patients and 80 of 81 nonelderly patients had a diagnosis of primary insomnia. Seven elderly patients and 1 nonelderly patient had a diagnosis of insomnia associated with physical disorders.

**Table 2:** Comorbid psychiatric disorders in insomnia patients with psychiatric disorders

**Comorbid Psychiatric Disorder, n (%)**	**Elderly**	**Nonelderly**
	**(n = 81)**	**(n = 80)**
Major depressive disorder	49 (60.5)	59 (73.8)
Generalized anxiety disorder	11 (13.6)	6 (7.5)
Dysthymic disorder	10 (12.3)	7 (8.8)
Agoraphobia	5 (6.2)	6 (7.5)
Panic disorder	4 (4.9)	7 (8.8)
Social phobia	4 (4.9)	5 (6.3)
Psychotic disorder	3 (3.7)	3 (3.8)
Obsessive-compulsive disorder	1 (1.2)	8 (10.0)
Neurosis	1 (1.2)	2 (2.5)

Among patients with insomnia and comorbid psychiatric disorders, the most common concomitant medications were psychoneurotics, anxiolytics, and peptic ulcer medications (Table 3). The most common concomitant medications among those without psychiatric disorders were analgesics, antipruritics, astringents, anti-inflammatory agents, antihypertensives, and antihyperlipidemic agents.

**Table 3:** Concomitant medications

**Concomitant Medication, n (%)**	**Insomnia With Psychiatric Disorders**		**Insomnia Without Psychiatric Disorders**	
	**Elderly**	**Nonelderly**	**Elderly**	**Nonelderly**
	**(n = 81)**	**(n = 80)**	**(n = 83)**	**(n = 81)**
Psychoneurotic	47 (58.0)	52 (65.0)	8 (9.6)	4 (4.9)
Sedative, anxiolytic	31 (38.3)	30 (37.5)	7 (8.4)	3 (3.7)
Antipeptic ulcer	31 (38.3)	19 (23.8)	17 (20.5)	11 (13.6)
Analgesic, antipruritic, astringent, anti-inflammatory	29 (35.8)	15 (18.8)	28 (33.7)	10 (12.3)
Antihyperlipidemic agents	27 (33.3)	5 (6.3)	25 (30.1)	8 (9.9)
Antihypertensive agents	27 (33.3)	1 (1.3)	25 (30.1)	10 (12.3)
Vasodilators	23 (28.4)	3 (3.8)	20 (24.1)	7 (8.6)
Antipyretic, analgesic,anti-inflammatory	21 (25.9)	22 (27.5)	22 (26.5)	21 (25.9)
Cold and cough preparations	10 (12.3)	14 (17.5)	9 (10.8)	6 (7.4)
Other anti-allergy	8 (9.9)	14 (17.5)	10 (12.0)	4 (4.9)
Oriental medicines	18 (22.2)	9 (11.3)	16 (19.3)	8 (9.9)
Expectorant	7 (8.6)	5 (6.3)	13 (15.7)	10 (12.3)
Antitussive	6 (7.4)	6 (7.5)	11 (13.3)	8 (9.9)
Antibiotics^a^	9 (11.1)	8 (10.0)	9 (10.8)	8 (9.9)

^a^ Acting mainly on gram-positive and gram-negative bacteria.

### Treatment compliance

Most elderly patients and all nonelderly patients had an overall treatment compliance rate of >70%, measured as the number of days on which the patient took study medication since the previous assessment. Treatment compliance rates through Week 4 of the treatment period (the day of dose escalation judgment) were >70% for the majority of elderly patients and for all nonelderly patients. No differences in treatment compliance were observed between treatment groups or between groups with and without psychiatric disorders.

### Safety

The safety analysis set included 164 elderly patients and 161 nonelderly patients. Overall, 81.5% of elderly patients in the 1-mg group and 79.5% of those in the 2-mg group reported at least 1 adverse event. The most frequently reported adverse events in this group were dysgeusia and nasopharyngitis (Table 4). The rate of adverse events was 79.5% (1-mg group) and 81.0% (2-mg group) for patients with psychiatric disorders and was 83.3% (1-mg group) and 78.0% (2-mg group) in patients without psychiatric disorders. In nonelderly patients, the rate of adverse events was 82.1% in the 2-mg group and 87.0% in the 3-mg group. The most common adverse events were similar to those in elderly patients (Table 4). Among nonelderly patients, ≥1 adverse event was reported by 79.1% (2-mg group) and 91.9% (3-mg group) of patients with psychiatric disorders and by 85.4% (2-mg group) and 82.5% (3-mg group) of patients without psychiatric disorders, respectively. Overall, no adverse events occurred at a substantially higher rate among the elderly and nonelderly populations in patients with psychiatric disorders versus those without psychiatric disorders. Overall, 5 adverse events led to discontinuation by 5 elderly patients and 9 adverse events led to discontinuation by 8 nonelderly patients. Four adverse events in elderly patients (anxiety, ECG abnormality, headache, vertigo) and 5 in nonelderly patients (abnormal hepatic laboratory value, dysgeusia, gastric ulcer, malaise, somnolence) were judged by the investigator to be possibly related to study medication. Adverse events leading to discontinuation but assessed by investigators as unrelated to study medication included appendicitis, bone fracture, depression (n = 2), and urticaria,

**Table 4:** Adverse events occurring in ≥2% of patients in either age group

**Adverse Event, n (%)**	**Elderly**		**Nonelderly**	
	**1 mg**	**2 mg**	**2 mg**	**3 mg**
	**(n = 81)**	**(n = 83)**	**(n = 84)**	**(n = 77)**
Dysgeusia	15 (18.5)	23 (27.7)	36 (42.9)	44 (57.1)
Nasopharyngitis	14 (17.3)	18 (21.7)	22 (26.2)	14 (18.2)
Headache	4 (4.9)	5 (6.0)	3 (3.6)	1 (1.3)
Somnolence	4 (4.9)	2 (2.4)	3 (3.6)	6 (7.8)
Upper respiratory tract infection	4 (4.9)	3 (3.6)	2 (2.4)	4 (5.2)
Back pain	4 (4.9)	3 (3.6)	2 (2.4)	1 (1.3)
Blood creatine phosphokinase increased	4 (4.9)	1 (1.2)	3 (3.6)	2 (2.6)
Thirst	3 (3.7)	0 (0.0)	2 (2.4)	2 (2.6)
Glucose urine present	3 (3.7)	2 (2.4)	1 (1.2)	1 (1.3)
Dizziness	1 (1.2)	4 (4.8)	0 (0.0)	1 (1.3)
Pharyngitis	1 (1.2)	2 (2.4)	3 (3.6)	1 (1.3)

The only adverse event considered by the investigator to be at least possibly related to study drug with a rate of ≥5% in elderly patients was dysgeusia (18.5% in the 1-mg group and 27.7% in the 2-mg group). In nonelderly patients, adverse events considered at least possibly related to study drug with a rate of ≥5% were dysgeusia (42.9%) in the 2-mg group and dysgeusia (57.1%) and somnolence (7.8%) in the 3-mg group. Nasopharyngitis, occurred in 20% of elderly patients and 22% of nonelderly patients but was judged by the investigators to have no causal relationship with eszopiclone administration in all cases.

Most adverse events were rated by the investigator as mild or moderate in severity; adverse events rated as severe occurred in 2.5% (2 of 81) of elderly patients in the 1-mg group and 0.0% (0 of 83) in the 2-mg group. Severe adverse events that occurred in the 1-mg group included sick sinus syndrome and loss of consciousness in 1 patient and major depressive disorder in a second patient. For nonelderly patients, adverse events rated as severe occurred in 1.2% (1 of 84) of patients in the 2-mg group and 2.6% (2 of 77) in the 3-mg group. Severe adverse events that occurred in nonelderly patients were acute myocardial infarction and heat illness, each occurring in 1 patient in the 2-mg group, and atopic dermatitis occurring in 1 patient in the 3-mg group. In addition, there was 1 completed suicide, noted below as a serious adverse event (SAE). In both elderly and nonelderly patients, all severe adverse events were considered by the investigator to be unrelated to study drug.

Twelve SAEs occurred in 10 patients (7 events in 6 elderly patients and 5 events in 4 nonelderly patients), all of which were assessed by investigators as unrelated to study drug. Among elderly patients, SAEs were reported in 3 patients in the 1-mg group with psychiatric disorders (aggravated type 1 diabetes mellitus in 1 patient, aggravated major depressive disorder in 1 patient, and both sick sinus syndrome and loss of consciousness in 1 patient), 1 patient in the 2-mg group with psychiatric disorders (oesophagitis haemorrhagic), and 2 patients in the 2-mg group without psychiatric disorders (angina pectoris in 1 patient, and left calculus ureteric in another patient). In nonelderly patients, SAEs other than death occurred in 2 patients in the 2-mg group with psychiatric disorders (appendicitis in 1 patient and acute myocardial infarction and heat illness in another patient) and in 1 patient in the 3-mg group without psychiatric disorders (left clavicle fracture). There was 1 death during the study; a 31-year-old man in the 3-mg group with psychiatric disorders died by suicide 22 days after the start of treatment. In addition to insomnia, the patient had depression, dysthymia, irritable bowel syndrome, and hepatic stenosis.

There were no clinically important changes from baseline in vital signs or laboratory parameters in nonelderly or elderly patients. Abnormal changes in ECG were not observed in nonelderly patients but were observed in 2.5% of elderly patients (2 of 79) in the 1-mg group and in 1.2% (1 of 83) in the 2-mg group.

Based on the Questionnaire of Drug Dependence, there was no evidence of psychological or physical drug dependency in any of the treatment groups. Furthermore, there were no significant differences in dependency questionnaire findings between treatment groups.

### Efficacy

#### Self-report sleep diary

**Figure 3:**
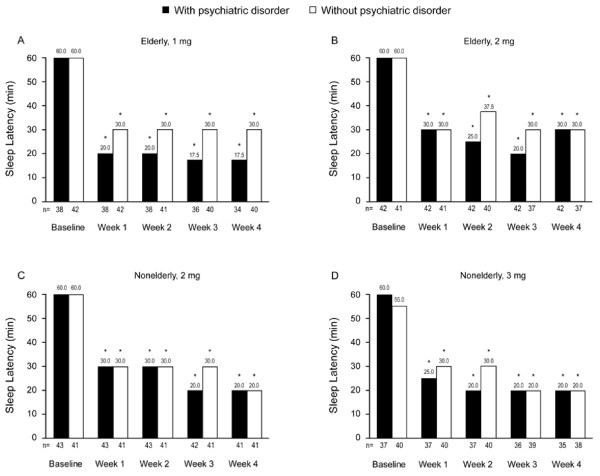
**Median values for sleep latency at baseline and at Weeks 1 through 4.** Median sleep latency (baseline; Week 1 through 4) were obtained in elderly patients receiving eszopiclone 1 mg (A) or 2 mg (B) and in nonelderly patients receiving eszopiclone 2 mg (C) or 3 mg (D). *P < 0.001 versus baseline for all treatment groups at all time points.

Eighty-eight percent of elderly patients and 83% of nonelderly patients reported improvement in both SL and TST at Week 4 compared with baseline. SL and TST outcomes were similar in those with versus those without comorbid psychiatric disorders. Eszopiclone 1 mg and 2 mg significantly decreased median SL from 60 minutes at baseline to 20 or 30 minutes at Week 1 in elderly insomnia patients with and without psychiatric disorders, and the effects were sustained through Week 4 (*P* < 0.001 for all comparisons) (Figures 3A and 3B). Similarly, eszopiclone 2 mg and 3 mg significantly improved SL in nonelderly patients with and without psychiatric disorders at all assessment points (Weeks 1, 2, 3, and 4; *P* < 0.001 for all comparisons) (Figures 3C and 3D).

**Figure 4:**
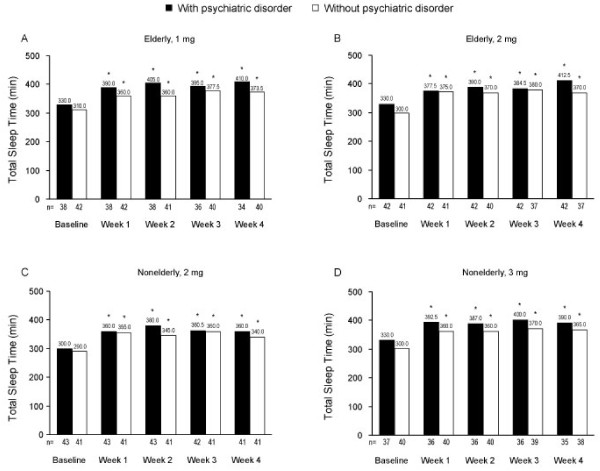
**Median values for total sleep time at baseline and at Weeks 1 through 4.** Median total sleep time (baseline; Weeks 1 through 4) were obtained in elderly patients receiving eszopiclone 1 mg (A) or 2 mg (B) and in nonelderly patients receiving eszopiclone 2 mg (C) or 3 mg (D). *P < 0.001 versus baseline for all treatment groups at all time points.

In elderly and nonelderly insomnia patients, both with and without psychiatric disorders, TST was significantly greater at Week 1 than at baseline, and the increased TST persisted through Week 4 (*P* < 0.001 for all comparisons; Figure 4). There were no statistically significant differences between dose groups on prespecified ANOVA models of SL or TST at any time point.

Measures of WASO and NA at Weeks 1 to 4 were significantly improved from baseline for all patient subgroups (*P* < 0.05 for all comparisons). In addition, quality of sleep (*P* < 0.05 for all comparisons) and depth of sleep (*P* < 0.05 for all comparisons) at Weeks 1 to 4 were also significantly improved from baseline for all patient subgroups. Inter-group comparisons (1 mg vs 2 mg in the elderly; 2 mg vs 3 mg in the nonelderly) revealed no significant differences between different doses on ANOVA models of WASO and NA at all assessment points (Weeks 1 to 4).

In both elderly and nonelderly patients, significant improvement in self-reported daytime sleepiness and daytime ability to function were observed at all assessment points during the first treatment period (*P* < 0.01 for all comparisons, elderly patients; *P* < 0.05 for all comparisons, nonelderly patients). Significant improvements (*P* < 0.05) from baseline in daytime sleepiness and daytime ability to function were observed in paired *t*-tests for the final week of the treatment period (Week 4) for elderly and nonelderly patients with and without psychiatric disorders, with the exception of daytime sleepiness in nonelderly patients with psychiatric disorders in the 3-mg group who experienced small but not statistically significant improvement.

#### Judgment of dose increase and efficacy at week 24

The investigators evaluated the appropriateness of dose escalation per patient for the second treatment period in a double-blind manner based on the criteria described in the study procedures. As a result, 6 patients in the elderly group and 11 patients in the nonelderly group were confirmed to be eligible for 1-mg uptitration (Table 5). Of these patients, 4 elderly patients receiving 1 mg eszopiclone and 7 nonelderly patients receiving 2 mg eszopiclone underwent uptitration (1 mg), whereas other patients who had been treated with higher doses in each group received a placebo tablet (ie, alternative to 1 mg eszopiclone) in addition to their current assigned treatment for the second treatment period.

**Table 5:** Judgment regarding 1 mg uptitration at Week 4

**Dose in First Treatment Period, Subgroup (n)**	**Improved SL and TST at Week 4, n (%)**	**Not Eligible, n**	**Eligible**^**a**^**, n**
**Elderly**			
1 mg, with psychiatric disorders (n = 34)	29 (85.3)	32	2
2 mg, with psychiatric disorders (n = 42)	39 (92.9)	41	1
1 mg, without psychiatric disorders (n = 40)	34 (85.0)	38	2
2 mg, without psychiatric disorders (n = 36)	32 (88.9)	35	1
Total elderly (n = 152)	134 (88.2)	146	6
**Nonelderly**			
2 mg, with psychiatric disorders (n = 41)	36 (87.8)	39	2
3 mg, with psychiatric disorders (n = 35)	28 (80.0)	32	3
2 mg, without psychiatric disorders (n = 41)	31 (75.6)	36	5
3 mg, without psychiatric disorders (n = 38)	33 (86.8)	37	1
Total nonelderly (n = 155)	128 (82.6)	144	11

^a^ Patients in the elderly 2-mg group and the nonelderly 3-mg group for whom dose escalation would be appropriate were given a 1-mg placebo tablet.

SL = sleep latency; TST = total sleep time.

**Figure 5:**
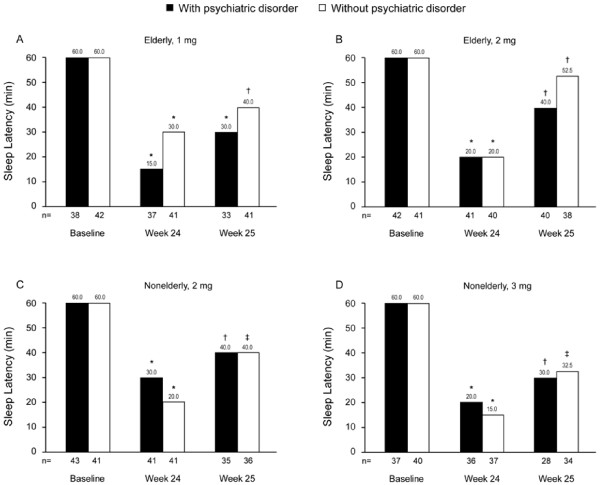
**Median values for sleep latency at baseline, Week 24, and Week 25.** Median sleep latency (baseline; Weeks 24 and 25) were obtained in elderly patients receiving eszopiclone 1 mg (A) or 2 mg (B) and in nonelderly patients receiving eszopiclone 2 mg (C) or 3 mg (D). For the patients who discontinued study treatment, data at final administration of study drug were handled as Week 24 data, and data at 1 week after final administration of study drug were handled as Week 25 data (last observation carried forward [LOCF]). *P < 0.001 versus baseline; ^†^*P* < 0.05 versus baseline; ^‡^*P* < 0.01 versus baseline.

At Week 24 (LOCF), significant improvement from baseline was noted for all patient subgroups in SL (Figure 5) and TST (nonelderly patients: median change of +60.0 min and +50.0 min for eszopiclone 2 mg and 3 mg, respectively; elderly patients: median change of +60.0 min and +70.0 for eszopiclone 1 mg and 2 mg; *P* < 0.001 for all comparisons). Similarly, WASO, NA, quality of sleep, and depth of sleep were significantly improved in all patient subgroups (*P* < 0.01 for all comparisons). For measures of daytime sleepiness and daytime ability to function, improvement was significant (*P* < 0.05) for all subgroups, with the exception of small but not statistically significant improvement in daytime sleepiness among nonelderly patients with psychiatric disorders receiving 3 mg eszopiclone. There was no apparent evidence of rebound insomnia following eszopiclone discontinuation (Table 6). Significant improvement from baseline in SL was maintained during the follow-up period in all subgroups at all assigned doses of eszopiclone (*P* < 0.05 for all comparisons; Figure 5). During the follow-up period, TST and WASO demonstrated improvement or maintained the same status compared with baseline in both elderly and nonelderly patients, regardless of psychiatric comorbidity status.

**Table 6:** Sleep variables at baseline and Week 25 (after discontinuation of eszopiclone)

**Measure**	**Elderly**		**Elderly**		**Nonelderly**		**Nonelderly**	
	**1 mg**		**2 mg**		**2 mg**		**3 mg**	
	**Baseline**	**Week 25**	**Baseline**	**Week 25**	**Baseline**	**Week 25**	**Baseline**	**Week 25**
Sleep latency, min	n = 80	n = 74	n = 83	n = 78	n = 84	n = 71	n = 77	n = 62
Mean (SD)	67.8 (42.3)	50.8 (47.8)	76.2 (48.9)	70.7 (81.2)	75.5 (51.3)	65.4 (67.3)	70.8 (46.4)	61.5 (66.3)
Median	60.0	30.0	60.0	40.0	60.0	40.0	60.0	30.0
Total sleep time, min	n = 80	n = 74	n = 83	n = 78	n = 84	n = 71	n = 77	n = 62
Mean (SD)	311.0 (68.9)	340.0 (96.3)	299.3 (59.8)	328.7 (102.3)	282.0 (47.7)	315.3 (61.1)	312.4 (67.1)	336.3 (80.6)
Median	330.0	360.0	300.0	360.0	290.0	300.0	300.0	337.5
Wake time after sleep onset,								
min	n = 79	n = 74	n = 82	n = 77	n = 84	n = 71	n = 77	n = 62
Mean (SD)	70.8 (57.8)	46.0 (49.1)	77.2 (64.8)	50.8 (53.3)	70.3 (65.5)	28.0 (32.6)	50.2 (42.0)	35.7 (54.0)
Median	60.0	30.0	60.0	30.0	60.0	15.0	45.0	15.0
Number of awakenings	n = 80	n = 74	n = 83	n = 77	n = 84	n = 71	n = 77	n = 62
Mean (SD)	2.1 (0.9)	1.6 (1.0)	2.2 (1.0)	2.0 (1.1)	1.9 (1.0)	1.2 (1.0)	1.9 (1.4)	1.7 (1.7)
Median	2.0	2.0	2.0	2.0	2.0	1.0	2.0	1.0
Quality of sleep *	n = 80	n = 74	n = 83	n = 78	n = 84	n = 71	n = 77	n = 62
Mean (SD)	4.1 (1.8)	5.4 (2.3)	4.2 (1.8)	5.3 (2.5)	3.4 (1.2)	4.5 (2.2)	4.1 (1.7)	4.8 (2.8)
Median	4.0	5.0	4.0	5.0	3.0	4.0	4.0	5.0
Depth of sleep *	n = 80	n = 74	n = 83	n = 78	n = 84	n = 71	n = 77	n = 62
Mean (SD)	4.2 (1.9)	5.4 (2.3)	4.3 (2.1)	5.1 (2.6)	3.3 (1.2)	4.7 (2.1)	4.1 (1.8)	4.8 (2.7)
Median	4.0	5.0	4.0	5.0	3.0	4.0	4.0	5.0
Daytime sleepiness *	n = 80	n = 74	n = 83	n = 78	n = 84	n = 71	n = 77	n = 62
Mean (SD)	5.1 (2.2)	6.2 (2.3)	5.0 (1.9)	6.0 (2.4)	4.1 (1.7)	4.9 (2.3)	4.7 (2.1)	5.5 (2.6)
Median	5.0	6.0	5.0	6.0	4.0	5.0	5.0	6.0
Daytime ability to function *	n = 80	n = 74	n = 83	n = 78	n = 84	n = 71	n = 77	n = 62
Mean (SD)	5.6 (2.1)	6.7 (2.1)	5.6 (2.0)	6.4 (2.2)	4.5 (1.7)	5.6 (2.1)	4.9 (1.9)	5.9 (2.5)
Median	5.5	7.0	5.0	7.0	4.5	5.0	5.0	6.0

*: Assessed based on a numeric rating scale (0–10 scale, with higher values indicating more positive outcomes).

#### Short-form 36

Baseline scores for the Physical Component Summary of the SF-36 in elderly patients with psychiatric disorders were poor (40.9 and 41.1 for 1 mg and 2 mg, respectively; Table 7). Scores in nonelderly patients with and without psychiatric disorders and in elderly insomnia patients without psychiatric disorders ranged from 46.8 to 51.7. Eszopiclone did not significantly alter the Physical Component Summary scores in the 4 subgroups (Table 7).

**Table 7:** Change in SF-36 scores from baseline to final visit

**Dose, Subgroup (n at final visit)**	**Physical ComponentSummary**	**Mental ComponentSummary**	**Mental HealthDomain**
**Elderly**			
1 mg, with psychiatric disorders (n = 37)^a^			
Baseline	40.9 (15.9)	43.6 (9.9)	39.5 (12.2)
Final visit (LOCF)	42.4 (12.2)	50.8 (10.9)	47.3 (11.2)
Change from baseline	1.5 (10.4)	6.8 (11.2)*	7.3 (12.0)*
2 mg, with psychiatric disorders (n = 42)			
Baseline	41.1 (15.7)	44.7 (10.6)	41.5 (13.2)
Final visit (LOCF)	43.3 (12.4)	50.5 (9.0)	47.0 (11.3)
Change from baseline	2.3 (10.6)	5.8 (8.4)*	5.5 (8.8)*
1 mg, without psychiatric disorders (n = 42)			
Baseline	46.8 (10.3)	54.7 (7.2)	53.7 (8.7)
Final visit (LOCF)	48.4 (7.4)	57.5 (6.8)	57.0 (7.2)
Change from baseline	1.6 (7.0)	2.9 (7.2)^†^	3.3 (8.2)^†^
2 mg, without psychiatric disorders (n = 41)			
Baseline	51.7 (5.0)	55.3 (7.9)	54.6 (7.7)
Final visit (LOCF)	51.5 (6.2)	56.1 (7.4)	55.7 (7.0)
Change from baseline	−0.2 (5.3)	0.8 (7.5)	1.1 (8.7)
**Nonelderly**			
2 mg, with psychiatric disorders (n = 43)			
Baseline	47.1 (10.4)	38.3 (8.7)	37.7 (9.1)
Final visit (LOCF)	47.5 (9.8)	44.6 (9.7)	43.6 (10.1)
Change from baseline	0.4 (10.5)	6.3 (9.3)*	5.9 (9.8)*
3 mg, with psychiatric disorders (n = 36)^a^			
Baseline	49.4 (8.8)	39.8 (8.2)	39.5 (6.9)
Final visit (LOCF)	49.5 (11.0)	44.2 (8.3)	44.4 (10.1)
Change from baseline	−0.1 (8.9)	4.3 (9.2)^‡^	4.8 (11.3)^†^
2 mg, without psychiatric disorders (n = 41)			
Baseline	50.6 (8.0)	49.8 (7.6)	50.1 (9.1)
Final visit (LOCF)	51.7 (9.7)	50.8 (9.2)	50.7 (10.5)
Change from baseline	1.2 (8.6)	1.0 (9.6)	0.7 (12.0)
3 mg, without psychiatric disorders (n = 39)^a^			
Baseline	50.9 (11.4)	49.4 (7.9)	50.4 (7.8)
Final visit (LOCF)	52.5 (8.1)	51.2 (8.1)	51.9 (7.8)
Change from baseline	1.7 (10.8)	1.7 (5.9)	1.6 (7.3)

All values represent mean (SD). Norm-based scorings (50 points represents the mean national standard value of Japanese people, with a standard deviation of 10).

^a^ 1 discontinuation.

*P < 0.001, ^†^*P* < 0.05, ^‡^*P* < 0.01.

LOCF = last observation carried forward; SD = standard deviation.

Baseline scores for the Mental Component Summary in both elderly and nonelderly insomnia patients with psychiatric disorders were 38.3 to 44.7 (Table 7), which is below the Japanese national standard value of 50 points. Eszopiclone significantly improved Mental Component Summary scores by 4.3 to 6.8 at final visit (*P* < 0.01 for all comparisons; Table 7). Mental Component Summary scores in elderly and nonelderly insomnia patients without psychiatric disorders were 49.4 to 55.3 at baseline and either slightly improved with eszopiclone (*P* < 0.05) or remained within the same range. The mental health domain is 1of 8 SF-36 domains and is also 1of 5 components of the Mental Component Summary. Changes in mental health domain scores from baseline to final visit showed a similar tendency as the Mental Component Summary scores (Table 7).

## Discussion

This study in Japanese elderly and nonelderly adults with chronic insomnia, with and without comorbid psychiatric disorders, extended the favorable safety and tolerability profile of eszopiclone. The inclusion/exclusion criteria for this study were generally similar to criteria used in US studies [9-13,15-20]. The principal difference is that this Japanese study was heterogeneous in terms of age range and insomnia subtype, whereas separate US studies were used to evaluate elderly or non-elderly patients and patients with primary or comorbid insomnia [9-13,15-20]. In the present study, the most frequently reported adverse event was dysgeusia, which is a characteristic adverse event associated with eszopiclone treatment. Overall, the rates of adverse events that led to discontinuation of study treatment were relatively low, and no unexpected adverse events were noted from a safety viewpoint.

Eszopiclone significantly improved sleep variables (SL, TST, WASO, NA, sleep quality, and sleep depth) and daytime activity in elderly and nonelderly patients both with and without psychiatric disorders. These improvements were observed in the first week after initiating treatment with eszopiclone and were maintained over 4 weeks of treatment. There was no evidence of daytime impairment, and the numeric rating scale scores generally indicated subjective improvement from baseline in daytime sleepiness and daytime functioning. Otherwise, the benefits of eszopiclone related to sleep parameters and daytime activity were similar in elderly and nonelderly patients. Efficacy evaluations were performed at Week 24 as part of the assessment of rebound insomnia and demonstrated that improvements in sleep and daytime functioning were maintained over 24 weeks of treatment. No rebound insomnia was observed after discontinuation of eszopiclone treatment. These observations confirm that the benefits of eszopiclone reported in US clinical studies of nonelderly [9-11] and elderly [12,13] patients with chronic insomnia (improvements in SL, TST, WASO, NA, sleep quality, sleep depth, and daytime activity) apply to the Japanese population.

The pharmacokinetics of eszopiclone have previously been compared in Japanese and Caucasian healthy adult and elderly subjects (unpublished data). The results showed that the pharmacokinetic and safety profiles of eszopiclone were similar in the Japanese population and the Caucasian population (unpublished data). Therefore, it is anticipated that ethnic factors are unlikely to influence the pharmacokinetics, efficacy, and safety of eszopiclone. In the current study, there was a lack of dose differentiation between 1-mg and 2-mg eszopiclone in elderly patients and between 2 mg and 3 mg eszopiclone in nonelderly patients with regard to all sleep studies (Weeks 1 to 4). These observations differ from the dose responses observed in US studies [9,11,13]. A 2-week study of eszopiclone in elderly patients with chronic insomnia reported that the higher dose (2 mg) significantly improved SL, TST, WASO, quality of sleep, and depth of sleep relative to placebo, but the lower dose (1 mg) improved SL but not TST, WASO, quality of sleep, or depth of sleep versus placebo [13]. Similarly, in the 6-week study of eszopiclone (2 mg and 3 mg versus placebo) in nonelderly adults with chronic insomnia, both doses improved SL and TST, but the 2-mg dose did not improve WASO [9]. This finding is supported by a polysomnography crossover study in nonelderly adults that demonstrated improvement in WASO with eszopiclone 3 mg but not 2 mg [11]. The reasons underlying the apparent difference in dose-responsiveness between Japanese and US populations are not well understood. The heterogeneous patient population in this study, which included both patients with primary and comorbid insomnia, does not seem to be an explanatory factor. In US studies, eszopiclone was effective for sleep induction and maintenance at the same dose (3 mg for non-elderly patients, 2 mg for elderly patients) regardless of the presence of psychiatric comorbidity [9-13,15,16,18-20].

Insomnia commonly presents comorbidly with psychiatric disorders, particularly anxiety disorders and depression [23-26]. Recent studies have shown that eszopiclone improves both sleep variables and psychiatric symptoms in nonelderly insomnia patients with depression receiving fluoxetine [19], and patients with generalized anxiety disorder receiving escitalopram oxalate [20]. The current study examined the mental and physical health effects of long-term eszopiclone treatment in elderly and nonelderly insomnia patients with or without a psychiatric disorder. Eszopiclone significantly improved Mental Component Summary scores on the SF-36 among elderly and nonelderly patients with comorbid psychiatric illness; median scores were below the national standard value at baseline and increased to approximately the national standard level following treatment. Further, eszopiclone produced statistically significant improvement on the Mental Health Domain among patients with psychiatric comorbidities, and the 1-mg dose of eszopiclone also significantly improved Mental Health Domain scores among elderly patients without comorbid psychiatric illness. These findings suggest that eszopiclone can improve quality of life in patients with insomnia associated with a psychiatric disorder.

A limitation of the current study was that it was not placebo- or active-controlled. As discussed earlier, inclusion of a placebo arm was considered unacceptable because of the long-term nature of the study and the presumed distress associated with untreated insomnia, particularly in patients with comorbid psychiatric or physical disorders. However, the lack of a placebo group limits the strength of conclusions regarding the efficacy of eszopiclone and the absence of a rebound effect. In addition, statistical tests comparing Week 24 and 25 were not performed; it is therefore difficult to assess changes in sleep parameters upon discontinuation of eszopiclone. Also, the potential effects of dose escalation on efficacy of eszopiclone could not be adequately assessed, because an increased dose was needed by only a small proportion of patients in the study. Furthermore, the ability to generalize results from this study to the general Japanese population is limited by the exclusion of patients with certain disorders (eg, PTSD, history of drug abuse, or medication-related insomnia). Although use of medications with the potential to modify sleep was restricted until the primary efficacy evaluation at Week 4, some potentially sedating drugs (eg, anxiolytics not indicated for insomnia) were permitted after Week 4 and may have affected subsequent assessments of the efficacy and tolerability of eszopiclone. Other limitations include the lack of polysomnographic recording of sleep parameters and objective assessment of daytime functioning, cognitive function, and behavioral adverse effects (eg, potential carryover sedation), which may have been more sensitive ways of assessing for potential dose response effects of eszopiclone in this population.

## Conclusions

In conclusion, 24 weeks of treatment with eszopiclone in Japanese elderly and nonelderly patients with chronic insomnia was shown to be safe and well tolerated, with no evidence of rebound insomnia or dependency following discontinuation. Results of this study demonstrate benefits of eszopiclone on sleep variables in a clinically representative sample of the Japanese population. Further, this study shows for the first time that benefits are maintained over 24 weeks in both elderly and nonelderly patients, as well as in patients with and without comorbid psychiatric disorders. The safety of eszopiclone and effects on sleep variables and daytime activity are consistent with what was observed in the US pivotal studies. In contrast to US studies, there was no apparent difference between the lower (1 mg in elderly patients, 2 mg in nonelderly patients) and higher (2 mg in elderly patients, 3 mg in nonelderly patients) eszopiclone doses in the Japanese patients, suggesting that it is rational to start with the low doses (1 mg in elderly patients, 2 mg in nonelderly patients) and titrate upward if needed.

## Abbreviations

ECG, Electrocardiogram; LOCF, Last observation carried forward; NA, Number of awakenings; PTSD, Posttraumatic stress disorder; SAE, Serious adverse event; SD, Standard deviation; SF-36, 36-item Short Form (SF-36) Health Survey; SL, Sleep latency; TST, Total sleep time; WASO, Wake time after sleep onset.

## Competing interests

This study was sponsored by Eisai Co., Ltd., Tokyo, Japan. NU serves as a consultant for Eisai, MSD, and Takeda. AK and TT are full-time employees of Eisai Co., Ltd.

## Authors’ contributions

All authors contributed in the concept and design, acquisition of data, analysis and interpretation of data, drafting of the manuscript, or revising it critically for important intellectual content. All authors read and approved the final manuscript.

## References

[B1] Roth T Insomnia: definition, prevalence, etiology, and consequences J Clin Sleep Med 2007 3 5 Suppl S7 S10 17824495 PMC1978319

[B2] State-of-the-Science Conference Statement on manifestations and management of chronic insomnia in adults NIH Consens Sci Statements 2005 22 1 30 17308547

[B3] Doi Y Epidemiologic research on insomnia in the general Japanese populations Nihon Rinsho 2009 67 1463 1467 19768925

[B4] Neubauer DN The evolution and development of insomnia pharmacotherapies J Clin Sleep Med 2007 3 5 Suppl S11 S15 17824496 PMC1978321

[B5] Hartikainen S Lönnroos E Louhivuori K Medication as a risk factor for falls: Critical systematic review J Gerontol A Biol Sci Med Sci 2007 62 1172 1181 10.1093/gerona/62.10.1172 17921433

[B6] Owen RT Eszopiclone: an update on its use in insomnia Drugs Today 2011 47 263 275 21573250 10.1358/dot.2011.47.4.1590792

[B7] Nutt DJ Stahl SM Searching for perfect sleep: the continuing evolution of GABAA receptor modulators as hypnotics J Psychopharmacol 2010 24 1601 1612 10.1177/0269881109106927 19942638

[B8] Lunesta® (eszopiclone) tablets 1 mg, 2 mg, 3 mg: US prescribing information 2010 Sunovion Pharmaceuticals Inc, Marlborough, MA, USA

[B9] Zammit GK McNabb LJ Caron J Amato DA Roth T Efficacy and safety of eszopiclone across 6-weeks of treatment for primary insomnia Curr Med Res Opin 2004 20 1979 1991 10.1185/174234304X15174 15701215

[B10] Krystal AD Walsh JK Laska E Caron J Amato DA Wessel TC Roth T Sustained efficacy of eszopiclone over 6 months of nightly treatment: results of a randomized, double-blind, placebo-controlled study in adults with chronic insomnia Sleep 2003 26 793 799 14655910 10.1093/sleep/26.7.793

[B11] Erman MK Zammit G Rubens R Schaefer K Wessel T Amato D Caron J Walsh JK A polysomnographic placebo-controlled evaluation of the efficacy and safety of eszopiclone relative to placebo and zolpidem in the treatment of primary insomnia J Clin Sleep Med 2008 4 229 234 18595435 PMC2546455

[B12] McCall WV Erman M Krystal AD Rosenberg R Scharf M Zammit GK A polysomnography study of eszopiclone in elderly patients with insomnia Curr Med Res Opin 2006 22 1633 1642 10.1185/030079906X112741 16968566

[B13] Scharf M Erman M Rosenberg R Seiden D McCall V Amato D Wessel TC A 2-week efficacy and safety study of eszopiclone in elderly patients with primary insomnia Sleep 2005 28 720 727 16477959 10.1093/sleep/28.6.720

[B14] Foley D Ancoli-Israel S Britz P Walsh J Sleep disturbances and chronic disease in older adults: results of the 2003 National Sleep Foundation Sleep in America Survey J Psychosom Res 2004 56 497 502 10.1016/j.jpsychores.2004.02.010 15172205

[B15] Ancoli-Israel S Krystal AD McCall WV Schaefer K Wilson A Claus R Rubens R Roth T A 12-week, randomized, double-blind, placebo-controlled study evaluating the effect of eszopiclone 2 mg on sleep/wake function in older adults with primary and comorbid insomnia Sleep 2010 33 225 234 20175406 10.1093/sleep/33.2.225 PMC2817909

[B16] Walsh JK Krystal AD Amato DA Rubens R Caron J Wessel TC Schaefer K Roach J Wallenstein G Roth T Nightly treatment of primary insomnia with eszopiclone for six months: effect on sleep, quality of life, and work limitations Sleep 2007 30 959 968 17702264 10.1093/sleep/30.8.959 PMC1978384

[B17] Roth T Walsh JK Krystal A Wessel T Roehrs TA An evaluation of the efficacy and safety of eszopiclone over 12 months in patients with chronic primary insomnia Sleep Med 2005 6 487 495 10.1016/j.sleep.2005.06.004 16230048

[B18] McCall WV Blocker JN D’Agostino R Kimball J Boggs N Lasater B Haskett R Krystal A McDonald WM Rosenquist PB Treatment of insomnia in depressed insomniacs: effects on health-related quality of life, objective and self-reported sleep, and depression J Clin Sleep Med 2010 6 322 329 20726279 PMC2919661

[B19] Fava M McCall WV Krystal A Wessel T Rubens R Caron J Amato D Roth T Eszopiclone co-administered with fluoxetine in patients with insomnia coexisting with major depressive disorder Biol Psychiatry 2006 59 1052 1060 10.1016/j.biopsych.2006.01.016 16581036

[B20] Pollack M Kinrys G Krystal A McCall WV Roth R Schaefer K Rubens R Roach J Huang H Krishnan R Eszopiclone coadministered with escitalopram in patients with insomnia and comorbid generalized anxiety disorder Arch Gen Psychiatry 2008 65 551 562 10.1001/archpsyc.65.5.551 18458207

[B21] Diagnostic and statistical manual of mental disorders, 4th edition, text revision (DSM-IV-TR) 2000 American Psychiatric Press, Inc., Washington, DC

[B22] Kurihara M Jimbo M Hirose T Asano J Endo S Fujiya Y Hada H Haga M Hasue I Higuchi T Karasumi H Motomura H Naito T Nakamura R Nakauchi M Narita S Noguchi T Oyama K Okada M Okazaki Y Sasaki K Takahashi K Takei H Takeyama K Watanabe J Double-blind comparison of clinical effects of ID 540 (fludiazepam) diazepam and placebo on psychoneurotic patients and a tentative draft of dependency questionnaire [Japanese] Rinsho Hyoka (Clinical Evaluation) 1977 5 341 368

[B23] Ohayon MM Roth T Place of chronic insomnia in the course of depressive and anxiety disorders J Psychiatr Res 2003 37 9 15 10.1016/S0022-3956(02)00052-3 12482465

[B24] Roth T Jaeger S Jin R Kalsekar A Stang PE Kessler RC Sleep problems, comorbid mental disorders, and role functioning in the national comorbidity survey replication Biol Psychiatry 2006 60 1364 1371 10.1016/j.biopsych.2006.05.039 16952333 PMC1894656

[B25] Neckelmann D Mykletun A Dahl AA Chronic insomnia as a risk factor for developing anxiety and depression Sleep 2007 30 873 880 17682658 10.1093/sleep/30.7.873 PMC1978360

[B26] Buysse DJ Angst J Gamma A Ajdacic V Eich D Rössler W Prevalence, course, and comorbidity of insomnia and depression in young adults Sleep 2008 31 473 480 18457234 10.1093/sleep/31.4.473 PMC2279748

